# Reproduction and pathogenesis of short beak and dwarfish syndrome in Cherry Valley Pekin ducks infected with the rescued novel goose parvovirus

**DOI:** 10.1080/21505594.2022.2071184

**Published:** 2022-05-03

**Authors:** Jianye Wang, Yu Wang, Yonglin Li, Yuehua Gao, Yufeng Li, Zhiwei Jiang, Guoqiang Zhu, Xiaobo Wang

**Affiliations:** a College of Veterinary Medicine, Yangzhou University, Yangzhou, Jiangsu, China; b Jiangsu Co-Innovation Center for Important Animal Infectious Diseases and Zoonosis, Yangzhou, Jiangsu, China; c Institute of Poultry Science, Shandong Academy of Agricultural Sciences, Jinan, Shandong, China

**Keywords:** Goose parvovirus, short beak and dwarfish syndrome, reproduction, pathogenesis, rescue

## Abstract

Since the outbreak of short beak and dwarfish syndrome (SBDS) in Cherry Valley Pekin ducks in China, novel goose parvovirus (NGPV) has been isolated. Till now, little is known about the NGPV pathogenesis toward Cherry Valley Pekin ducks. Besides, due to detection of duck circovirus co-infection in SBDS clinical cases, whether sole NGPV infection can reproduce all the typical symptoms of SBDS remains unclear. In this study, based on the NGPV isolate SDJN19, an infectious plasmid clone pJNm containing the entire SDJN19 genome was constructed. Transfection of pJNm in embryonated duck eggs resulted in generation of the infectious virus carrying the genetic marker, named rJNm. rJNm infection of 2-day-old Cherry Valley Pekin ducks reproduced all the typical signs of SBDS, including beak atrophy, tongue protrusion, and growth retardation. rJNm can infect Cherry Valley Pekin ducks through the horizontal transmission route, and the infected ducks exhibited the characteristic SBDS symptoms. A high level of serum precipitation antibodies (above 5log_2_) were induced in the surviving ducks, however, high viral loads were still detected in the duck organs, suggesting persistent NGPV infection in ducks. By incorporating the homologous Rep1 and VP1 gene from classical GPV, two chimeric viruses rJN-cVP1 and rJN-cRep1 were generated. Duck infection tests revealed that the non-structural protein Rep1 played a crucial role in the NGPV pathogenicity. The present result lays a solid foundation for further exploring how the Rep protein contributes to the NGPV pathogenesis.

## Introduction

In 2015, short beak and dwarfism syndrome (SBDS) emerged in Cherry Valley Pekin ducks (a strain of Pekin duck) in China [1,2], thereafter, other breeds of ducks, including mule duck, partridge duck, and sheldrake duck, were also affected [3,4]. In 2019, SBDS emerged in Egypt and Poland, where mule ducks and Cherry Valley Pekin ducks were infected [5,6]. SBDS is a contagious disease of ducks, characterized by growth retardation, short beak, tongue protrusion, lameness, unwillingness to move, and diarrhea [7]. The morbidity of SBDS is 10%~30% and the mortality is less than 5% [1]. SBDS was first reported to occur in mule ducks (a hybrid of Pekin duck and Muscovy duck) in France in the 1970s [7]. The etiological agent of SBDS was identified to be a new lineage of goose parvovirus, namely novel goose parvovirus (NGPV) [5,7]. NGPV possesses characteristic nucleotide and amino acid differences relative to the classical goose parvovirus (GPV) [8].

GPV is placed under the species *Anseriform dependoparvovirus 1* in the genus *Dependoparvovirus* of the *Parvoviridae* family [9]. GPV has a single-stranded linear genome of approximately 5.1 kb, which is flanked by identical inverted terminal repeats (ITR) [10,11]. ITR consists of about 442 nucleotides, which is the longest among the *Parvoviridae* subfamily. The GPV genome contains two ORFs, the left encoding non-structural protein Rep and the right encoding the structural protein Cap. Through alternative pre-mRNA splicing, the Rep ORF produces the Rep1 protein and several low molecular masses of Rep proteins [12]. Rep protein can bind ITR and is involved in genome replication, packaging, viral rescue from plasmid vector, and transactivation with the downstream P41 promoter [13,14]. By splicing of pre-mRNAs and selective usage of initiation codons, the Cap ORF generates three structural proteins, namely VP1, VP2, and VP3, which share a common carboxyl terminal but different amino terminal [12,15].

Until now, little is known about the pathogenesis of NGPV toward Cherry Valley Pekin ducks. Furthermore, due to the finding of duck circovirus co-infection in the SBDS clinical cases [16,17], whether sole NGPV infection in Cherry Valley Pekin duck can reproduce all the typical symptoms of SBDS remains unclear. In this study, based on the NGPV isolate SDJN19, an infectious plasmid clone pJNm containing the whole genome of SDJN19 was constructed. Transfection of pJNm resulted in rescue of the infectious virus carrying a genetic marker. The infection test with the rescued virus rJNm demonstrated that sole NGPV infection of Cherry Valley Pekin ducks was sufficient to reproduce all characteristic signs of SBDS. NGPV can infect ducks via the horizontal transmission route and establish a persistent infection state in ducks, irrespective of high levels of serum precipitation antibodies induced after infection. Furthermore, two chimeric viruses were generated, in which the original Rep1 or VP1 gene was replaced by the counterpart from classical GPV. The duck infection test based on the chimeric viruses indicated that the Rep1 protein, but not VP1, played a critical role in the NGPV pathogenicity.

## Materials and methods

### Virus propagation

Strain SDJN19, which was isolated from Cherry Valley Pekin ducks manifesting SBDS symptoms in Shandong province in 2019 [8]. The viral stock, stored at ‒80°C in the form of allantoic fluid, was 1:30 diluted with sterile saline and supplemented with penicillin (1000 IU/ml) and streptomycin (1000 µg/ml). The viral dilution was used to inoculate 9-day-old embryonated Cherry Valley Pekin duck eggs via the allantoic cavity route, and these eggs were continuously incubated at 37.8°C. The embryos, which died in 24 hours were discarded, and the remaining eggs were candled three times daily. The dead embryos were picked out and cooled at 4°C for 4‒6 h, then the allantoic fluid was pooled and stored at ‒20°C until use.

### Virus purification and DNA extraction

Approximately 300 ml of allantoic fluid was centrifuged at 4,000 × g for 15 min, and the clarified supernatant was transferred into a clean container and chloroform was added with a volume ratio of 3:1. The mixture was drastically vibrated for 2 min, and centrifuged at 6,000 × g for 15 min. The upper aqueous phase was collected and subjected to refrigerated ultracentrifugation at 300,000 × g for 4 h (SW32Ti rotor, Beckman, USA). The supernatant was thoroughly discarded and the pellet was resuspended in 5 ml TE buffer (50 mM Tris, 20 mM EDTA, pH 8.0). The SDS-Proteinase K incubation method was used to extract viral nucleic acids. Briefly, 500 µl of the concentrated virus was added with a final concentration of 1% SDS and 400 µg/ml Proteinase K, and incubated at 50°C for 2 h in a constant temperature bath. The mixture was extracted with phenol-chloroform-isoamylol (25:24:1) and chloroform-isoamylol (24:1). The viral DNAs in the upper aqueous phase were pelleted with alcohol and dissolved in 30 µl STE (10 mm Tris, 1 mm EDTA, 100 mm NaCL, pH 8.0). The single-stranded DNAs were heated at 95°C for 5 min, then slowly annealed to 50°C resulting in formation of double-stranded DNA (dsDNA).

### Construction of the plasmid clone pJN

Based on the endonuclease site analysis, the NGPV genome has a single *Nco*I site at nucleotide position 3806, which was utilized in the genome cloning. A linker containing the *Nco*I site was synthesized and inserted into the plasmid pBluescript II SK (pBSK) between *Hin*dIII and *Eco*RI, resulted in replacement of the original *Eco*RV site with *Nco*I. The modified plasmid was named pBSKN.

**Figure 1.**
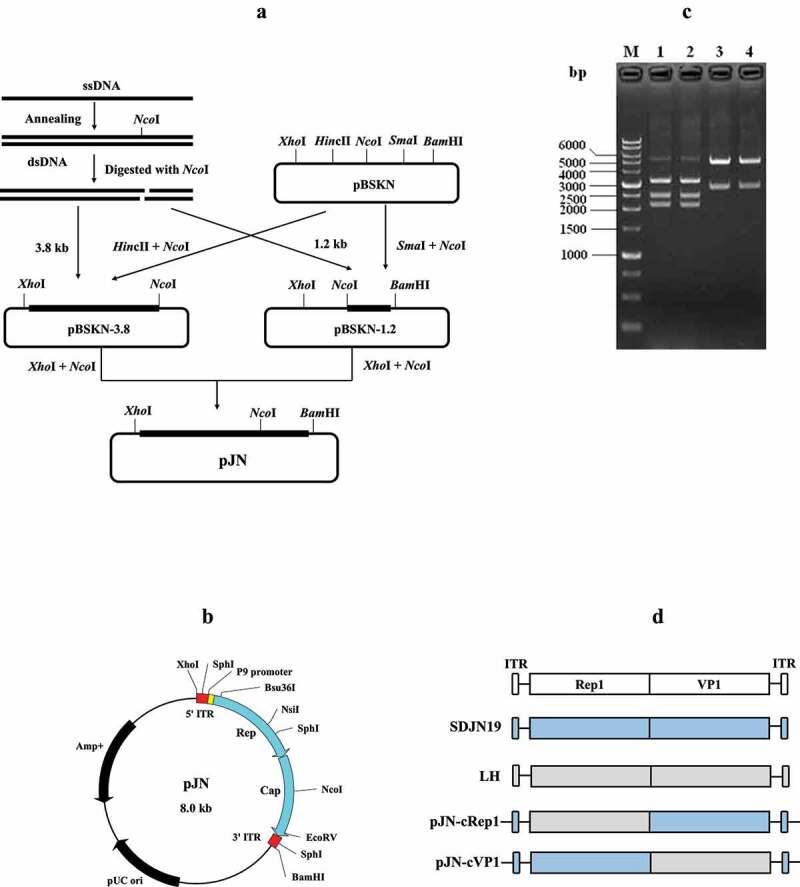
Construction and characterization of plasmids. (a) the strategy for constructing the infectious plasmid clone pJN containing the entire genome of the NGPV strain SDJN19. (b) Illustration of pJN harboring the SDJN19 genome. (c) Characterization of pJN by restriction endonuclease digestion. 1,2: Digestion with *Sph*I produced 3.3 kb, 2.5 kb and 2.2 kb DNA fragments; 3,4: Double digestion with *Xho*I and *Bam*HI produced a 3.0 kb vector and 5.0 kb genomic molecule. (d) the diagram depicting construction of the chimeric viruses. the original Rep1 or VP1 gene in plasmid pJN was replaced by the counterpart from the classical GPV strain LH through overlap PCR and usage of suitable restriction endonuclease sites.

The viral dsDNAs were digested with the restriction endonuclease *Nco*I, generating the 3.8 kb and 1.2 kb DNA fragments. The left 3.8 kb fragment was ligated with the linearized pBSKN plasmid predigested with *Nco*I and *Hin*cII, and the ligated product was transformed into competent cells of the Sure strain *E. coli*. The positive clone was named pBSKN-3.8. Similarly, the right 1.2 kb fragment was ligated with the *Nco*I- and *Sma*I-linearized pBSKN, generating the plamid pBSKN-1.2. The plasmids pBSKN-3.8 and pBSKN-1.2 were further digested with *Nco*I and *Xho*I, and the resulting 3.8-kb inserted fragment and the 4.2-kb backbone fragment were gel-purified and ligated. The ligation product was transformed into the competent cells of SURE strain and the positive clone was named pJN (Figure 1(a)).

### Rescue of infectious NGPV from pJnm carrying the genetic marker

To differentiate the rescued virus from the parental strain, a genetic marker was introduced in the plasmid pJN. Briefly, through overlap PCR, a synonymous nucleotide mutation (A→G) was engineered in the *Nde*I site within the VP3 gene region. The engineered plasmid pJN carrying the genetic marker was named pJNm. pJNm was purified using the TIANpure Mini Plasmid Kit (Tiangen, Pekin, China). pJNm was premixed with Lipofectamine 2000 (Invitrogen, Carlsbad, USA) at a ratio of 1:2.5 (μg:μl). The transfection mixture was injected into the chorioallantoic membranes of 9-day-old embryonated Cherry Valley Pekin duck eggs (2.0 μg per egg). Simultaneously, transfection of the vector plasmid pBSKN was performed as the control. When the embryos died from infection of the rescued virus after 48 h post-transfection, their allantoic fluid was pooled. The allantoic fluid was diluted 1:50 in sterile saline and passaged in 9-day-old embryonated Cherry Valley Pekin duck eggs twice. The genome of the rescued virus (named after rJNm below) was amplified using PrimeSTAR Max DNA polymerase (Takara, Dalian, China), and the overlapping fragments were purified and subjected to direct sequencing.

### Titer measurement of rJNm

The median embryo lethal dose (ELD_50_) experiment was conducted to compare the titer difference between rJNm and the parental strain SDJN19. Briefly, the allantoic fluid of each virus was diluted in sterile saline containing penicillin (1000 U/ml) and streptomycin (1000 mg/ml) to give a 10-fold dilution series between 10 ^−1^ and 10 ^−7^. For each dilution, 0.2 ml was inoculated into the allantoic cavity of each of five 9-day-old embryonated Cherry Valley Pekin duck eggs. These eggs were continually incubated and candled until the seventh day. The ELD_50_ value was calculated by the method of Reed and Muench [18].

### rJNm infection of Cherry Valley Pekin ducks

2-day-old susceptible Cherry Valley Pekin ducklings were divided into two groups. Twenty-two ducklings in the infection group were injected subcutaneously in the neck with rJNm containing 2.5 × 10^5^ ELD_50_. Twenty-two Ducklings in the control group were injected with 0.5 ml of the allantoic fluid from embryonated 14-day-old duck embryos. Both groups of ducks were accommodated in isolators, and food and water were provided *ad libitum*. These ducks were monitored for 32 days. At 7, 14, 21, 28 days post-infection (dpi), body weight, beak width, and length were measured. If death occurred, the postmortem examination was performed.

### Viral isolation from the infected ducks

Liver, spleen, and ileum tissues of the dead ducklings or the surviving ducks after euthanizing were sampled and homogenized with sterile PBS. The homogenates were clarified by centrifugation at 12,000×*g* for 5 min, and the supernatants were transferred and passed through a 0.22-µm-pore-diameter filter membrane. The filtrates were supplemented with penicillin (1000 IU/mL) and streptomycin (1000 µg/mL) and used to inoculate susceptible 9-day-old embryonated Cherry Valley Pekin duck eggs. If the embryos died after 48 h, their allantoic fluids will be pooled and used to extract DNAs as the templates for PCR amplification using primers targeting the NGPV VP1 gene. A .8 kb DNA fragment covering the introduced nucleotide mutation site was amplified and recovered for sequencing after electrophoresis analysis. To exclude duck circovirus infection occurred during the observation period, primers targeting to the conserved region of duck circovirus genotype 1 and 2 were designed. The upstream primer is 5′-CTGCCGCCCTTGAG/AGAGTC-3′, and the downstream primer is 5′-CCGAGTAACCGTCCCACCA-3′.

### Histopathological assessment

At 31 days post-infection, two living ducks were randomly chosen and euthanized. The internal organs were fixed in 10% neutral formalin at room temperature for 48 h, embedded in paraffin, and cut into 5-μm-thick sections. After deparaffinization, the sections were stained with hematoxylin and eosin (H&E). Pathological changes were observed under an Olympus microscope (Olympus, Tokyo, Japan) and evaluated by a pathologist.

### X-Ray and microcomputed tomography analysis of bone tissue

The leg and wing bone tissues of the above-used ducks were analyzed by X-ray using YEMA ClearXvet DR50 (Varian, Salt Lake, USA). Duck bone microstructure was evaluated using Skyscan1174 X-Ray Microtomograph (Micro CT) (Bruker, Belgium). Briefly, two ducks in the infection group and control group were euthanized in CO_2_ at 21 dpi, respectively. The duck tibias were dissociated and scanned with the following parameters: voltage, 50 kW; electric current, 800 μA; resolution, 30.2 μm per pixel. After scanning, the regions of interest (ROIs) were selected and the *N*-Recon software was used for 3D image reconstruction. Bone mineral density (BMD), bone volume/total volume (BV/TV), trabecular number (Tb.N), trabecular thickness (Tb.Th), trabecular separation (Tb.Sp) and the structural model index (SMI) were analyzed by CT-AN software.

### Viral load analysis of the infected ducks

Viral loads in the internal organs (Liver, spleen, and ileum) of the dead and surviving ducks were analyzed by absolute quantitative real-time PCR (qPCR) using the ChamQ universal SYBR qPCR master mix (Vazyme, Nanjing, China). A 1.4-kb Rep1 gene fragment was amplified and cloned into the pMD19T vector (Takara, Dalian, China), generating the plasmid pMD-Rep1.4 which was used as the standard plasmid template in the qPCR. A set of qPCR primers targeting the conserved region of NGPV and GPV were designed using the Primer 5.0 software. The upstream and the downstream primers are 5′-CAATGTTGGTCTTCCCGGTGG-3′ and 5′-AGTATCCTGTGCGGCTGGGTG-3′, respectively. A 95-bp gene fragment can be specifically amplified from the standard plasmid template, the NGPV-infected duck tissues, but not from the normal duck tissue samples. All the assays were performed in triplicate in an ABI 7500 real-time PCR system (Applied Biosystems, CA, USA) according to the manufacturer’s protocol.

To compare whether the dead ducks harbored the similar viral load levels with the deceased goslings due to classical GPV infection, the gosling infection test was performed. Briefly, eight 2-day-old goslings free of maternal GPV precipitation antibodies were infected with the virulent GPV strain LH (GenBank accession number KM272560). As in the rJNm infection test, the same viral dose (2.5 × 10^5^ ELD_50_) was used to inoculate each gosling subcutaneously in the neck. All of the eight goslings died at 4―5 days post-infection, and their internal organs (liver, spleen, and ileum) were collected and used for viral loads analysis.

### Horizontal transmission test of rJNm

To elucidate whether rJNm can effectively invade the Cherry Valley Pekin ducks via the horizontal transmission route, the horizontal transmission experiment was performed. Three groups, including the infection group, the horizontal contact group, and the mock control group, were set in the experiment. Each group contained nine 2-day-old Cherry Valley Pekin ducks and was raised in different cages. The infection group and the horizontal contact group cages were housed in a separate room and were about 10 cm apart. The control group was placed in another room. Air can flow freely within the room and between cages. The infection group was inoculated subcutaneously with rJNm at a dose of 2.5 × 10^5^ ELD_50_. All ducks in the three groups were allowed free access to water and feed. Clinical signs in ducks were monitored and recorded for a total of 32 days. Body weight, beak length, and width of three groups were measured at 7, 14, 21, 28 days post-infection. At the end of the experiment, the ducks were sacrificed by CO_2_ asphyxiation. Postmortem examination was performed, and the liver, spleen, and ileum tissues were collected for viral loads analysis and isolation.

The serum antibody titers of the surviving ducks were measured by agar gel precipitation (AGP) test. Antibody titers were expressed as the log_2_ of the reciprocal of the highest dilution of serum producing a precipitation line. The allantoic fluid containing viral particles of strain SDJN19 was concentrated and used as the precipitation antigen.

### Role of the virally coded proteins in NGPV pathogenicity

Based on the parental plasmid pJN, two chimeric plasmids pJN-cVP1 and pJN-cRep1 were constructed by overlap PCR and usage of unique restriction endonuclease sites. The infectious plasmid pLH containing the whole genome of the classical GPV strain LH was previously constructed in our lab [19]. The chimeric plasmids pJN-cVP1 and pJN-cRep1 incorporated the VP1 and Rep1 gene from pLH, respectively (Figure 1(d)). Both plasmids were propagated in Sure strain of *E.coli*. Each purified plasmid was used to transfect five 10-day-old embryonated goose eggs as above described in the pJNm transfection experiment. If death of goose embryos commences after 48 h post-transfection, the allantoic fluid is pooled. The pooled allantoic fluid was 1:10 diluted with sterile saline and passaged in another five 10-day-old embryonated goose eggs. The second passages of both chimeric viruses were quantitated by qPCR and used for the infection experiment.

Twenty-four 2-day-old Cherry Valley Pekin ducks were randomly divided into three groups (eight ducks per group), two challenge groups and a control group. Ducks in each challenge group were injected subcutaneously in the neck with the same dose of chimeric virus containing 2.5 × 10^10^ viral particles. The control group was injected with the equal volume of the allantoic fluid from 14-day-old embryonated duck eggs. These ducks were observed for 32 days, and body weight and beak length at 7, 14, 21, 28 days were recorded. Gross clinical signs were divided into 4-grade severities: no change, mild, moderate, and marked with scores of 0, 1, 2, and 3, respectively.

### Statistical analysis

The data were expressed as mean ± standard deviation (SD). GraphPad Prism software version 6.01 (GraphPad Software Inc., San Diego, CA, USA) was used for comparison of groups. P < 0.05 was considered statistically significant.

### Ethical statement

The procedure for inoculation of ducks, geese, and embryonated duck or goose eggs was approved by the Animal Care and Use Committee of Yangzhou University and performed in accordance with the “+Guidelines for Experimental Animals” of the Ministry of Science and Technology (Beijing, China). No specific permissions were required for these locations/activities. Throughout the experimental period, all the living or sick ducks were euthanized in CO_2_.

## Results

### Cloning of the SDJN19 genome and sequence analysis

The single-stranded DNA (ssDNA) was extracted from the concentrated virions. By annealing of ssDNA, the double-stranded DNAs (dsDNA) was obtained. Digestion of dsDNA with *Nco*I produced a 3.8-kb and a 1.2-kb DNA fragment, which were cloned into the pBSKN vector. The recombinant plasmids were transformed and propagated in competent *E. coli* cells of the Sure strain, which is suitable for cloning certain DNA segments that are unstable in conventional *E. coli* strains. Sequence analysis revealed that positive plasmid clones harbored intact ITR sequences without deletions. The entire SDJN19 genome consists of 5055 nucleotides, and shares 95% nucleotide homologies with the classical virulent strain LH of GPV (GenBank accession number KM272560). The ITR is composed of 418 nucleotides, among which the inside 379 nucleotides constitutes the palindromic hairpin structure and the outside 39 nucleotides forms the D region (Supplementary Figure S1).

### Construction of infectious plasmid clone pJnm

On the basis of the SDJN19 genome subclones, the 1.2 kb and 3.8 kb fragments were combined, generating the recombinant plasmid pJN (Figure 1(b)). Digestion of pJN with *Xho*I and *Bam*HI produced a 5.0-kb NGPV genomic molecule and a 3.0-kb pBSKN vector backbone. The SDJN19 genome has three *Sph*I sites, two in the middle loop of ITR and one in the coding region. Digestion of pJN with *Sph*I produced 2.2 kb, 2.5 kb, and 3.3 kb fragments (Figure 1(c)). Through overlap PCR, a single nucleotide mutation as the genetic marker was successfully introduced within the VP3 gene region of pJN. The engineered plasmid was named pJNm.

### Rescue of pJnm in embryonated Cherry Valley Pekin duck eggs

After transfection of pJNm in 9-day-old embryonated Cherry Valley Pekin duck eggs, the embryo deaths commenced at 120 h, and all embryos expired at 136 h post-transfection. Hemorrhagic lesions were observed across the bodies, legs, wings, eyes, and necks (Suplementary Figure S2). In the control group, all of the five duck embryos survived by the seventh day post-transfection of the vector plasmid pBSKN, and no pathological change was observed in these embryos. The allantoic fluid was pooled from the dead duck embryos. The rescued virus was passaged in five 9-day-old embryonated Cherry Valley Pekin duck eggs, and the duck embryos died between 96 h and 120 h. The rescued virus was named rJNm.

The whole genome of rJNm was amplified using overlapping primer sets. Sequence analysis revealed that the rJNm genome displayed 100% nucleotide homology with the parental strain SDJN19, except for the introduced nucleotide mutation as the genetic marker. Comparison of ELD_50_ between rJNm and the parental strain was performed in 9-day-old susceptible embryonated Cherry Valley Pekin duck eggs. The result revealed that rJNm had an ELD_50_ reaching 5 × 10^6.25^/ml, by contrast, the ELD_50_ of the parental strain SDJN19 was 5 × 10^5.38^/ml. Thus, rJNm possesses a similar titer in embryonated Cherry Valley Pekin duck eggs when compared to the parental strain SDJN19.

### rJNm infection of Cherry Valley Pekin ducks

**Figure 2.**
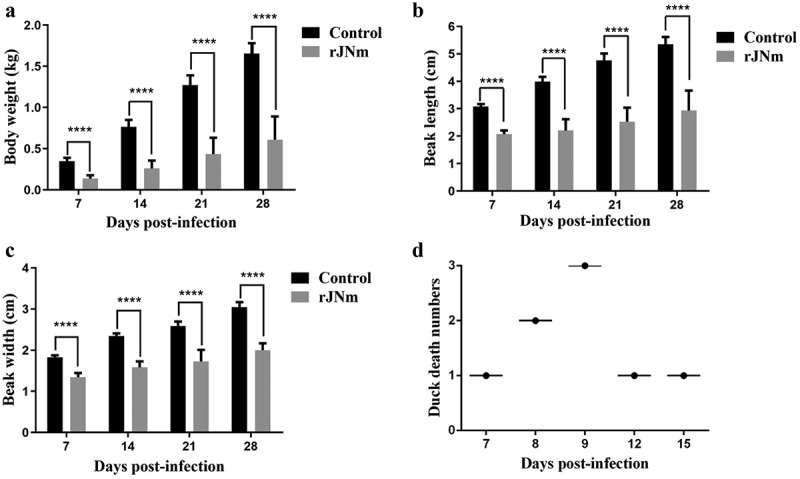
Influence of rJNm infection on body weight, beak development and survival of Cherry Valley Pekin ducks. (a) Measurement of body weight at 7, 14, 21, 28 days post-infection of 2-day-old ducks. (b) Measurement of beak length. (c) Measurement of beak width. ****, p < .0001, significant difference between the infection group and control group. (d) Death numbers of ducks observed at different days post-infection.

After inoculation with rJNm, the challenge group exhibited a clear tendency of growth retardation. The mean body weights of the infection group at 7, 14, 21, and 28 days post-infection (dpi) were 0.137, 0.261, 0.434, and 0.609 kg, whereas, the control group grew faster than the infection group since the first week, which had mean body weights of 0.349, 0.764, 1.271, and 1.656 kg at 7, 14, 21 and 28 dpi (Figure 2(a)). The significant differences (P < 0.0001) were present between the control and infection groups at each measuring point, implying that rJNm infection posed a serious effect on duck development. Beak length and width of the infected group at 7, 14, 21, 28 dpi were also significantly lower than the control group (P < 0.0001) (Figure 2(b–c)). As the disease progressed, death cases were produced at the early stage during the experimental period. A total of eight ducks died in the infection experiment and the mortality reached 40%. The first death case was found at 7 dpi, and the last death case occurred at 15 dpi (Figure 2(d)).

**Figure 3.**
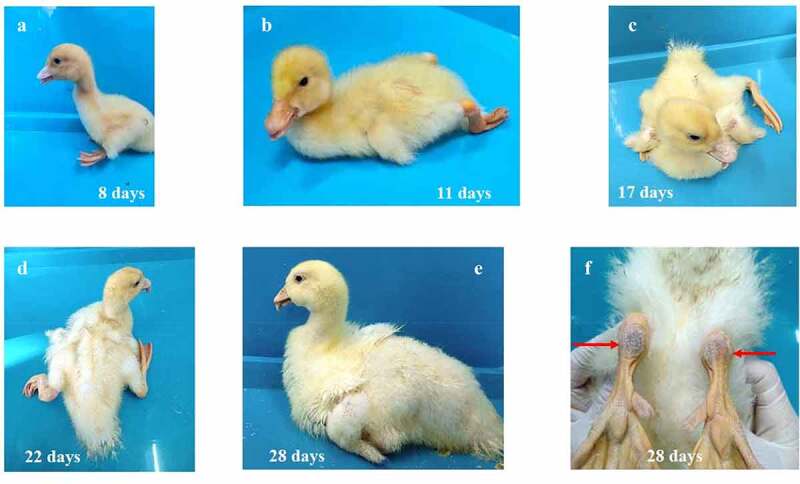
The gross clinical signs of Cherry Valley Pekin ducks at different days post-infection of rJNm. These signs included the inability to stand and move, lameness, one or both legs stretching back or outwards, protruding tongues, delayed molt (A-E), red and swollen metatarsus (F).

In addition to growth retardation, another prominent sign was bone development abnormality. Initially, the infected ducks were reluctant to move, crouching in most of the time. Later, the infected ducks had trouble in standing and walking, manifested by lameness, one or both legs stretching back, and red and swollen metatarsi (Figure 3). Due to shorter beaks, a high proportion of ducks exhibited a protruding tongue, which was clearly observed at about three-week post-infection. Other clinical signs observed in the living ducks included watery diarrhea, delayed malt and muffled quack. No nervous symptoms were observed in the infected ducks throughout the observation period.

The organ samples of the dead ducks, including liver and spleen, were used for viral isolation by inoculating 9-day-old embryonated Cherry Valley Pekin eggs. All the duck embryos died in ten days post-inoculation, and their allantoic fluids were harvested for PCR examination and sequencing. An 800 bp DNA fragment specific to NGPV VP3 gene was amplified from all samples. Sequence analysis of the amplified fragments revealed 100% homologies with the parental strain SDJN19, except for the introduced nucleotide mutation as the genetic marker. PCR amplification using the duck circovirus-specific primers revealed negative in all samples. The result demonstrated that deaths of ducks and manifestation of the typical SBDS signs in the infection test were attributed to rJNm infection.

### Gross lesions and histopathologic changes of the dead and living ducks

**Figure 4.**
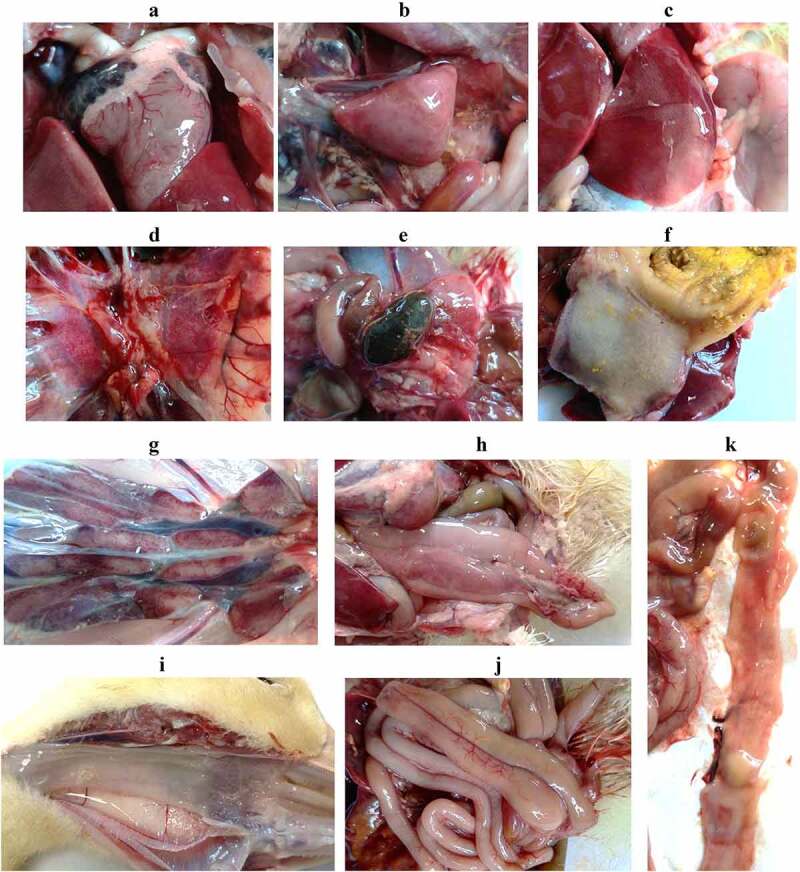
Gross lesions observed in the dead ducks after rJNm infection. Congestion was widely found in the internal organs, including heart, spleen, liver, lung, kidney, pancreatic gland (A-E, G-H). The gallbladder was full of dark green bile (E). The glandular stomach surface (F) and the esophageal mucosa (I) had excessive thick mucus attached. The duodenum was dilated and relaxed (J), and the mucosa became thinner, pale red, and had yellow inflammatory secretions attached (K).

After autopsy, the main pathological change of the dead ducks was congestion, which was found in the epicardial blood vessel, liver, kidney, spleen, lung, and pancreatic gland (Figure 4(a–d,g–h)). Besides, the gallbladder was full of dark green bile (Figure 4(e)). The surface of the glandular stomach and the esophageal mucosa had excessive thick mucus attached (Figure 4(f,i)). The duodenum was relaxed (Figure 4(j)), and the intestinal mucosa became thinner, pale red and had yellow inflammatory secretions attached (Figure 4(k)). The surviving ducks at 31 dpi were euthanized and their carcasses were examined by histology. Despite of the typical SBDS signs, the internal organs of these ducks showed neither gross pathological changes nor histological lesions.

### Viral loads analysis of the dead ducks

**Figure 5.**
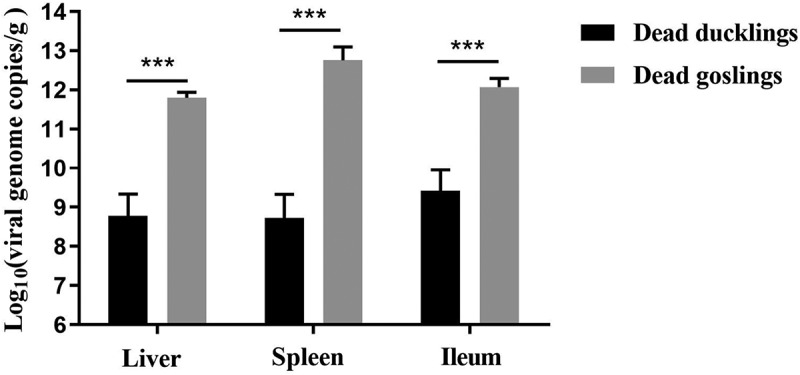
Viral loads in the internal organs of the dead ducks were determined by qPCR, which were compared with that of the deceased goslings after infection with the classical GPV strain LH. ***, significantly different, P<0.001.

Viral loads of the internal organs of the dead ducks were determined by qPCR and compared with that of the dead goslings, all of which died in five days post-challenge of the classical virulent strain of GPV. Mean viral loads in the livers, spleens, and ileums of the dead ducks reached 10^8.77^/g, 10^8.73^/g, and 10^9.42^/g, respectively. By contrast, higher viral loads were detected in the organs of the dead goslings (10^11.77^/g in livers, 10^12.71^/g in spleens, and 10^12.02^/g in ileums) (Figure 5). The viral loads of the dead ducks were about 1000-fold lower than that of the dead goslings, demonstrating that the NGPV has a differential propagation kinetics in Cherry Valley Pekin ducks.

### X-Ray and micro-CT analysis of duck bone tissues

**Figure 6.**
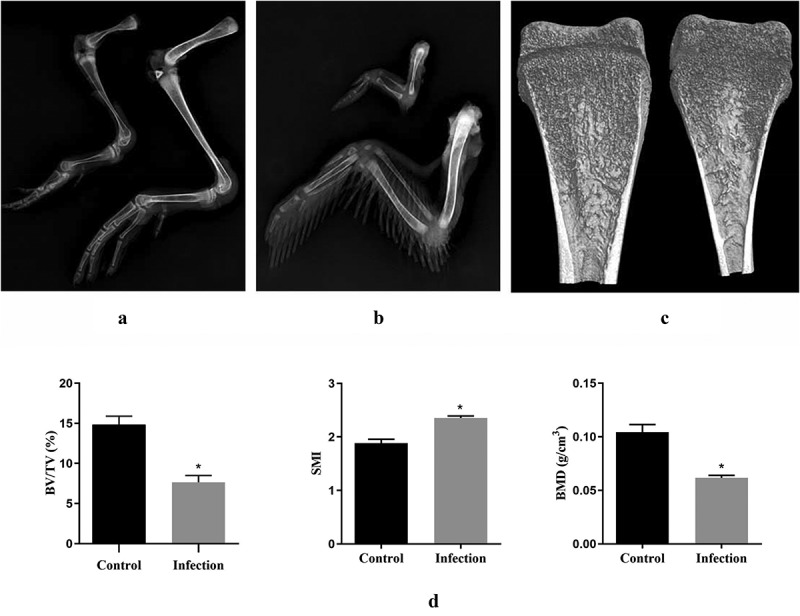
X-ray and micro CT analysis of duck bone tissues. (a) X-ray imaging of duck legs of the living ducks at 28 dpi. left, the rJNm-infected duck; right, the control duck. (b) X-ray imaging of duck wing. above, the rJNm-infected duck; below, the control duck. (c) Coronal section images of tibias of the living ducks at 21 dpi were created by micro CT. left, the control duck; right, the rJNm-infected duck. (d) Comparison of BV/TV, SMI and BMD of the tibias between the infected and control ducks at 21 dpi. *, significantly different, P < 0.05.

X-ray examination showed that rJNm infection caused serious effects on bone development of ducks. All bone components of the legs and wings were obviously shorter and narrower than the control group ducks at 28 days post-infection (Figure 6(a–b)). The tibia microstructure parameters were analyzed using micro-CT. The infected ducks exhibited a narrower marrow cavity (Figure 6(c)), and a lower BMD, BV/TV, and a higher SMI in comparison to the control group (P < 0.05) (Figure 6(d)). No significant differences were found in Tb.N, Tb.Th and Tb.Sp between groups. The result indicated that bone construction was seriously dampened in the rJNm-infected ducks.

### Horizontal transmission ability of rJNm

**Figure 7.**
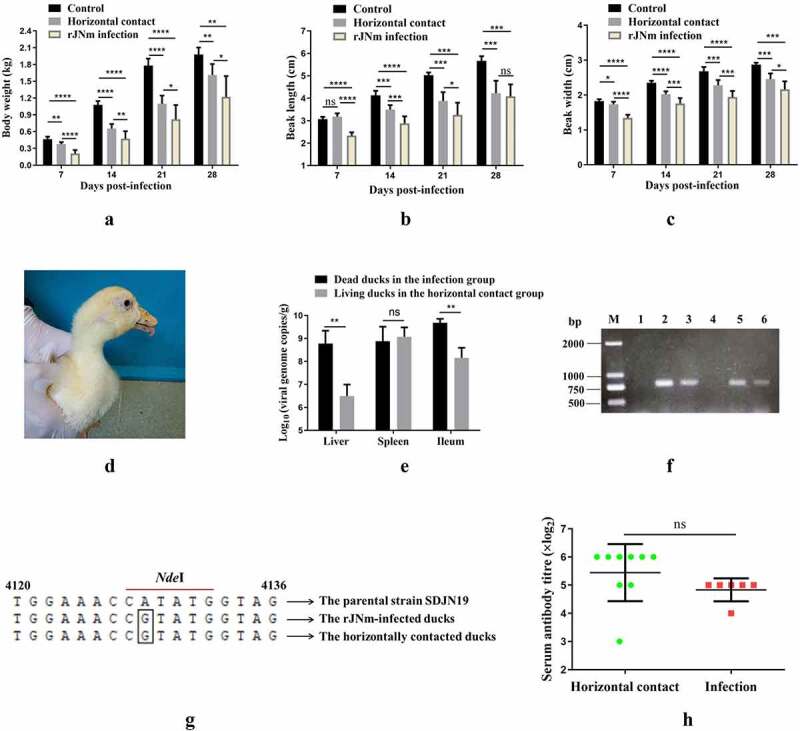
Performance of Cherry Valley Pekin ducks infected with rJNm via the horizontal transmission route. A-C: Comparison of duck body weight, beak length, beak width among the control group, the horizontal contact group and the rJNm infection group. *, p < 0.05; **, p < 0.01; ***, p < 0.001; ****, p < 0.0001; ns, no significant difference (p > 0.05). D: Tongue protrusion was clearly observed in the horizontal contact group ducks. E: Viral loads comparison of the internal organs between the dead ducks in the rJN infection group and the living ducks in the horizontal contact group at 31 days post-infection (dpi). F: 800-bp DNA fragments covering the genetic marker site were amplified from the spleens, ileums, but not livers of the living ducks in the horizontal contact group at 31 dpi. 1, 4: liver; 2, 5: spleen; 3, 6: ileum. G: Sequence analysis of the amplified 800-bp fragments revealed common nucleotide mutations (A→G) present in the dead ducks from the rJNm infection group and the living ducks from the horizontal contact group. H: Serum antibody titres of the living ducks in the horizontal contact group at 30 dpi were measured by the agar gel precipitation test and compared with the infection group. ns, no significant difference, p>0.05.

Body weights of the horizontal contact group were lower than the control group at 7, 14, 21, and 28 dpi (P < 0.01), but were higher than the infected group (P < 0.05) (Figure 7(a)). Beak lengths of the horizontal contact group were not significantly different from the control group at 7 dpi (P > 0.05), but were lower than the control group at 14, 21, 28 dpi (P < .01). At 28 dpi, the horizontal contact group had a mean beak length of 4.22 cm, which was similar to that of the infection group (4.08 cm) (P > 0.05), whereas, beak length of the control group reached 5.67 cm (Figure 7(b)). The beak widths of the horizontal contact group were always lower than the control group (Figure 7(c)). At 28 dpi, mean beak width of the horizontal contact group was 2.46 cm, which was lower than the control group (2.87 cm) (P < 0.001), but higher than the infection group (2.16 cm) (P < 0.05). During the observation period, no death occurred in the horizontal contact group, however, 6 out of 9 ducks exhibited protruding tongues (Figure 7(d)), which were obviously observed at 28 dpi. In addition, compared with the control group, ducks in the horizontal contact group cannot persistently stand and had muffled quacks.

The ducks in the horizontal contact group at 31 dpi had slightly higher viral loads (10^9.1^/g) in the spleens than the dead ducks from the infected group (10^8.9^/g), but the difference was not significant (P > 0.05) (Figure 7(e)). The viral loads in livers and ileums from the horizontal contact group reached 10^6.5^/g and 10^8.2^/g, which were lower than that of the dead ducks in the infected group (10^8.8^/g and 10^9.7^/g, respectively) (P < 0.01).

800-bp DNA fragments covering the genetic marker site were amplified from the spleen and ileum samples of the horizontal contact group using primers targeting to the NGPV VP1 gene (Figure 7(f)). Sequence analysis of the gel-recovered 800-bp fragments revealed 100% homologies with the parental strain SDJN19, except for the artificially introduced nucleotide mutation (A→G) as the genetic marker in rJNm (Figure 7(g)). The serum antibody titers of nine ducks in the horizontal contact group were measured at 31 dpi by agar gel precipitation (AGP) tests, averaging 5.38, by contrast, six living ducks in the infection group had a mean antibody titer of 4.83 (Figure 7(h)) (P > 0.05). No AGP antibody titer was detected in the control group ducks. The result demonstrated that high serum AGP titers were elicited in the horizontal contact group. No microscopic pathological lesions were detected by histology in the internal organs of the surviving ducks from the horizontal contact group at 29 dpi. In conclusion, the results demonstrated that NGPV possessed a strong horizontal transmission ability, and ducks infected with NGPV by the horizontal contact route still manifested the characteristic clinical signs of SBDS.

### Role of the coding proteins in NGPV pathogenicity

After plasmids transfection, two chimeric viruses, named rJN-cVP1 and rJN-cRep1, were obtained. Each of the chimeric viruses was 1:30 diluted with sterile saline and inoculated into twelve 9-day-old embryonated Cherry Valley Pekin duck eggs to evaluate the replication ability. These eggs were observed for 10 days and death times were recorded by hours. The result showed that a 100% mortality was observed in the rJN-cVP1 inoculation group and the mean death time (MDT) was 171 h; in contrast, a 67% mortality was observed in the rJN-cRep1 inoculation group and the MDT was 190 h. The result revealed that the Rep gene played a crucial role for NGPV in adapting to embryonated Cherry Valley Pekin duck eggs.

**Figure 8.**
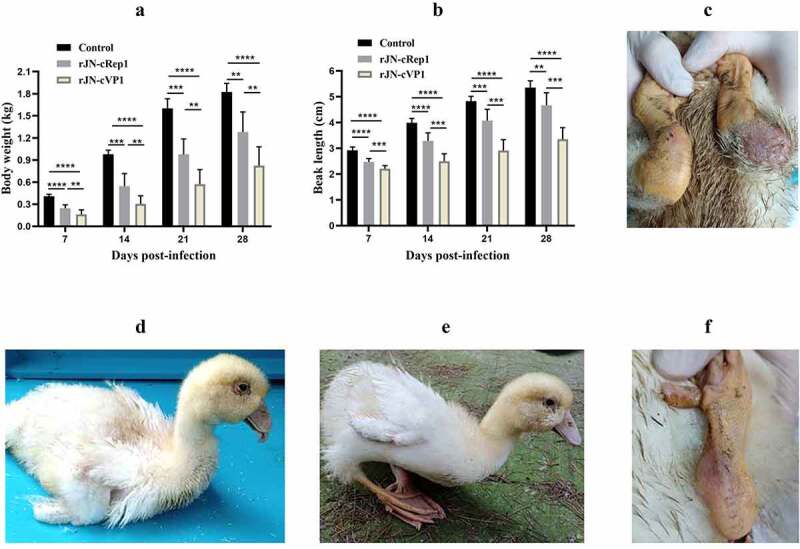
Comparison of body weights, beak lengths and other signs between therJN-cRep1 and the rJN-cVP1 infected ducks. (A-B) Comparison of body weights and beak lengths among the chimeric virus infection groups and the control group. *,p<0.05; **, p < 0.01; ***, p < 0.001; ****, p < 0.0001; (**C**) Two ducks in the rJN-cRep1 infection group showed red and swollen metatarsus at 28 dpi. (D-F) Ducks in the rJN-cVP1infection group showed typical signs of SBDS at 28 dpi, including dwarfishbody, tongue protrusion, lameness, delayed molt, and red and swollen metatarsi.

The pathogenicity of two chimeric viruses was evaluated in 2-day-old Cherry Valley Pekin ducks. Both challenge groups ducks exhibited lower body weights and shorter beaks than the control group (p  < 0.01), however, the rJN-cRep1 challenge group had heavier body weights and longer beaks than the rJN-cVP1 challenge group at 7, 14, 21, 28 dpi (p < 0.01) (Figure 8(a–b)). The clinical signs, including tongue protrusion, delayed molt, paralyzed legs, difficulty in standing and moving, were still visible in rJN-cVP1 infection group (Figure 8(d–f); Table 1). Besides, two ducks in the rJN-cVP1 infection group died at 9 and 18 dpi. By contrast, no death case was produced in the rJN-cRep1 infection group, in which only two ducks manifested red and swollen metatarsus (Figure 8(c)) and had trouble in standing and moving at the early infection stage (Table 1). The result demonstrated that the pathogenicity of rJN-cRep1 toward Cherry Valley Pekin ducks was severely impaired and the Rep1 protein, but not VP1, played a crucial role in NGPV pathogenicity and manifestation of the typical SBDS signs.

**Table 1.** Comparison of the clinical signs of Cherry Valley Pekin ducks post-infection of chimeric viruses

Clinical signs	Challenged virus	
	rJN-cVP1	rJN-cRep1
Tongue protrusion	+ + + (5/8)**	― (0/8)
Difficulty in standing and moving	+ + + (8/8)**	+ + + (2/8)
Delayed molt	+ + + (5/8) **	+ (2/8)
Death occurrence	2	0

Note: –, no change; +, mild; ++, medium; +++, marked. Data in parentheses indicate no. of ducklings with severities of clinical signs as indicated/no. of the infected ducks. ** indicate the significant difference (P < 0.01) between two infection groups.

## Discussion

Due to excellent meat yield and low-fat levels, there are a large rearing quantity for Cherry Valley Pekin ducks worldwide. Emergence of SBDS in Cherry Valley Pekin ducks has caused huge economic losses to duck producers. Since the outbreak of SBDS in China, NGPV was isolated from the clinical SBDS organ tissues [1,2]. However, NGPV co-infection with duck circovirus was also reported to be present in some field samples [16,17], hence, whether sole NGPV infection can reproduce all the typical SBDS symptoms in Cherry Valley Pekin ducks still confuses us. In the present study, through the reverse genetics technology, the infectious NGPV carrying the genetic marker was rescued and used in the infection and horizontal transmission test. The present result demonstrates that sole NGPV infection of Cherry Valley Pekin ducks is sufficient to reproduce all the clinical symptoms typical of SBDS.

In the infection test of Cherry Valley Pekin ducks, the first death case commenced at 7 dpi and the last death case commenced at 15 dpi. Time span between the first and last death case reached 8 days. Prior to death, these ducks presented characteristic appearances, including difficulty in standing and walking, leg paralysis, and inability to drink and eat. In contrast, classical GPV infection of goslings is characterized by acute infection and no characteristic signs were observed in these goslings prior to death. Therefore, different from classical GPV, NGPV has exhibited distinctive pathogenic characteristics in Cherry Valley Pekin ducks.

Viral load analysis of the dead and surviving ducks further demonstrated the distinctive pathogenicity of NGPV. In liver, spleen, and ileum, the dead ducks had viral loads ranging from 10^8^/g to 10^9^/g, in contrast, viral loads in the liver, spleen, and ileum of the dead goslings were nearly 1000-fold higher than the former, reaching 10^11^ ~ 10^12^/g. In addition, the viral loads in spleens of the living ducks from the horizontal contact group reached 10^9^/g by 31 days post-infection, which was basically at the same level as the viral loads in spleens of the dead ducks (10^8.9^/g). These results remind us that, unlike the Derzsy’s disease of geese, death of ducks in the infection test may not be directly due to hyperviremia. Analysis and comparison of duck bone tissue parameters demonstrated that NGPV infection produced serious effects on bone development, as was reflected in the clinical performance, including lameness, difficulty in moving, red and swollen metatarsi. Consequently, normal function of feeding and drinking were seriously weakened, which probably acted as an important factor leading to death of the infected ducks. Bone metabolism abnormality in human caused by the measles virus and canine distemper virus infection has been reported [20,21]. Hence, how NGPV infection disturbs the signaling pathway related to duck bone metabolism and affects bone development deserves to be thoroughly investigated.

NGPV also demonstrated a strong horizontal transmission ability in the horizontal transmission test. By 28 days after infection, most of ducks in the horizontal contact group showed typical signs of SBDS, including shorter beaks, tongue protrusion, poor body weights, and muffled quack, however, no death occurred. The experimental result is in agreement with the observation from the field, where the morbidity of SBDS in Cherry Valley Pekin duck flocks is comparatively high (10–30%), but the mortality is extremely low, generally below 5% [1,5,7].

The present study also demonstrated that NGPV can keep a persistent infection state in Cherry Valley Pekin ducks. Ducks in the horizontal contact group showed high levels of serum precipitation antibodies at 31 dpi. Despite the outcome, high viral loads were still detected from the spleens, ileums and livers of the living ducks. Especially in spleens, the living ducks had very similar viral load levels (~10^9^/g) with the dead ducks. PCR amplification further confirmed the presence of NGPV in the spleens and ileums, and the amplicon sequences were 100% identical to the parental strain SDJN19. These results demonstrated that high level of serum precipitation antibodies induced by NGPV infection was not sufficient to clear NGPV replication *in vivo*, leading to persistent infection. Among the members of the *Parvoviridae* family, Aleutian mink disease parvovirus (ADV) and B19 parvovirus are known to cause chronic and persistent infections in mink and human, respectively [22,23]. Therefore, how NGPV keeps the persistent infection state in Cherry Valley Pekin ducks deserves further investigation. Since Cherry Valley Pekin ducks grow very fast and are usually on the market in about 40 days of age, NGPV-infected ducks are likely to continually shed virus till marketing. As GPV is highly resistant to the natural environmental condition, in the “all-in-all-out” feeding mode, strict disinfection of feeding sites is of importance for controlling early NGPV infection and transmission.

Artificial infection of Cherry Valley Pekin ducks with classical GPV could not reproduce any symptom of SBDS, indicating that classical GPV is apathogenic to ducks (unpublished data). In terms of the encoding proteins Rep1 and VP1, there are 12 and 16 characteristic amino acid differences, respectively, between classical GPV and NGPV [8], however, which protein plays a dominant role in NGPV pathogenicity remains unknown. In this study, the rJN-cRep1 infection group still exhibited lower body weights and shorter beaks than the control group, however, no protruding tongues or death cases were produced, and most of the infected ducks freely moved. By contrast, in the rJN-cVP1 infection group, two death cases were found, and body weights and beak lengths were significantly lower than the control group and rJN-cRep1 infection group. In addition, tongue protrusion, bone deformity, and difficulty in standing and moving were observed to varying degrees at 28 dpi. Overall, the clinical signs typical of SBDS still can be reproduced in the rJN-cVP1 infection group, but not the rJN-cRep1 infection group. This result suggests that it is the Rep1 protein, but not VP1, that plays a dominant role in the NGPV pathogenicity and manifestation of typical SBDS signs. The Rep protein is multi-functional and is involved in genome replication and virion packaging by interacting with ITR [13,14]. It acts in trans with the P41 promoter and modulates the structural protein gene expression [24]. In addition, Rep proteins can manipulate inflammatory signaling pathways [25], induce host cell apoptosis [26–28], and exacerbate hepatocyte damage [29]. The 12 characteristic amino acid differences between NGPV and classical GPV are not evenly distributed across the Rep1 peptide chain, among which 8 amino acid sites are distributed near carboxyl terminus and 3 amino acid sites near amino terminus [8]. However, which mutated amino acid sites of Rep1 are closely related to the NGPV pathogenicity remains to be investigated.

Taken together, in this study, the infectious NGPV carrying the genetic marker was constructed and rescued. The bird infection and horizontal transmission tests demonstrated that sole NGPV infection of Cherry Valley Pekin ducks can reproduce all typical clinical signs of SBDS, and NGPV possesses a strong horizontal transmission ability. Despite the presence of high level of serum precipitation antibodies induced by NGPV infection, NGPV can keep the persistent infection state in Cherry Valley Pekin. Furthermore, the bird infection test based on the chimeric viruses showed that the non-structural protein Rep1, but not the structural protein VP1, played a key role in the NGPV pathogenicity and exhibition of the typical SBDS signs.

## Supplementary Material

Supplemental Material

## Data Availability

The datasets generated and/or analyzed during the current study are not publicly available due to the project is not finished yet but are available from the corresponding author on reasonable request.
