# Supplementary material for: Reproduction and pathogenesis of short beak and dwarfish syndrome in Cherry Valley Pekin ducks infected with the rescued novel goose parvovirus
Source: Virulence. 2022 May 3;13(1):844–58. doi: 10.1080/21505594.2022.2071184 (PMC9090291; doi:10.1080/21505594.2022.2071184)
Supplement: Supplemental Material [file KVIR_A_2071184_SM0123.zip › supplementary/Supplementary Figure S1.docx]

Supplementary Figure S1. Diagram of the 5′ ITR secondary structure of the SDJN19 strain. The nucleotide alterations relative to the classical GPV strain LH are highlighted with dark red, and nucleotide insertions are underlined. The D sequence comprises of 39 nucleotides and is denoted by circles above the letters, which is reversely complementary to the D′ sequence at the 3′ ITR. The bubble region consists of 43 nucleotides (nt position 169 to 211) and contains a *Sph*I site (GCATGC) in the middle loop.
