# Supplementary material for: Reproduction and pathogenesis of short beak and dwarfish syndrome in Cherry Valley Pekin ducks infected with the rescued novel goose parvovirus
Source: Virulence. 2022 May 3;13(1):844–58. doi: 10.1080/21505594.2022.2071184 (PMC9090291; doi:10.1080/21505594.2022.2071184)
Supplement: Supplemental Material [file KVIR_A_2071184_SM0123.zip › supplementary/Supplementary Figure S2.docx]

Supplementary Figure S2. Pathological changes of the Cherry Valley Pekin duck embryos that died between 120 h and 136 h after transfection of plasmid pJNm. Haemorrhagic lesions were observed in the bodies, legs, wings, eyes and necks (A). No pathological change was found in the control duck embryos at 7 days post-transfection of the vector plasmid pBSKN (B). The scale bar is 30 mm.
